# Reappraisal of visiting policies and procedures of patient’s family information in 188 French ICUs: a report of the Outcomerea Research Group

**DOI:** 10.1186/s13613-016-0185-x

**Published:** 2016-08-26

**Authors:** Maité Garrouste-Orgeas, Isabelle Vinatier, Alexis Tabah, Benoit Misset, Jean-François Timsit

**Affiliations:** 1 IAME, UMR 1137, Sorbonne Paris Cité, Paris Diderot University, 75018 Paris, France; 2 Outcomerea Research Group, 75020 Paris, France; 3 Medical-Surgical ICU, Les Oudaries Hospital, La Roche-Sur-Yon, France; 4 Department of Intensive Care Medicine, Royal Brisbane and Women’s Hospital, Brisbane, Australia; 5 Burns, Trauma and Critical Care Research Centre, University of Queesland, Brisbane, Australia; 6 Medical ICU, Charles Nicolle University Hospital, Rouen, France; 7 Medical ICU, Bichat University Hospital, Paris, France; 8 Service de médecine intensive et de réanimation, Groupe Hospitalier Paris Saint Joseph, 185 rue Raymond Losserand, 75014 Paris, France

**Keywords:** ICU, Family, Visiting policy, Information to family, Post-intensive care syndrome, Family information meeting, Access to information

## Abstract

**Background:**

The relatives of intensive care unit (ICU) patients must cope with both the severity of illness of their loved one and the unfamiliar and stressful ICU environment. This hardship may lead to post-intensive care syndrome. French guidelines provide recommendations on welcoming and informing families of ICU patients. We questioned whether and how they are applied 5 years after their publication.

**Methods:**

We conducted a large survey among French ICUs to evaluate their visiting policies and how information was provided to patient’s family. A questionnaire was built up by intensivists and nurses. French ICUs were solicited, and the questionnaire was sent to all participating ICUs, for being filled in by the unit medical and/or nursing head. Data regarding the hospital and ICU characteristics, the visiting policy and procedures, and the management of family information were collected.

**Results:**

Among the 289 French ICUs, 188 (65 %) participated. Most ICUs have a waiting room 118/188 (62.8 %) and a dedicated room for meeting the family 152/188 (80.8 %). Of the 188 ICUs, 45 (23.9 %) were opened on a 24-h-a-day basis. In the remaining ICUs, the time period allowed for visits was 4.75 ± 1.83 h (median 5 h). In ICUs where visiting restrictions were reported, open visiting was allowed for end-of-life situations in 107/143 (74.8 %). Children are allowed to visit a patient in 164/188 (87.2 %) regardless of their age in 97/164 (59.1 %) of ICUs. Families received an information leaflet in 168/188 (89.3 %). Information was provided to families through structured meetings in 149/188 (79.2 %) of ICUs at patient admission with participation of nurses and nursing assistants in 133/188 (70.4 %) and 55/188 (29.2 %), respectively. Information delivered to the family was reported in the patient chart by only 111/188 ICUs (59 %). Participation in care was infrequent.

**Conclusions:**

Although French ICUs do not follow the consensus recommendations, slow progress exists compared to previous reports. Implementation of these recommendations is largely needed to offer better welcome and information improvement. Further studies on that topic would enable evaluating remaining obstacles and increasing caregivers’ awareness, both critical for further progresses on that topic.

**Electronic supplementary material:**

The online version of this article (doi:10.1186/s13613-016-0185-x) contains supplementary material, which is available to authorized users.

## Background

Families of intensive care patients expressed psychological distress during the ICU stay of their relatives. The discovery of an unknown environment with often poorly understood medical information, combined with feeling fear about the prognosis uncertainty and the sometimes limited possibility to be present at patient’s bedside, contributes to anxiety and depression onset rapidly after ICU admission. Numerous studies described the feelings of family’s members during an ICU stay [1–3]. The French FAMIREA group described that 65 % and 35 % of the families developed anxiety and depression symptoms, respectively, within the few days after admission [4] and, for 33 % of them, a post-acute stress symptoms within 3 months after discharge [5]. Having a loved one dying in ICU can be responsible of complicated grief in 52 % of the relatives [6] or of heavy burden [7]. The importance of such consequences has been recently described under the term of post-intensive care syndrome-family [8, 9]. Numerous approaches have been described to limit these consequences on families’ members, such as the development of family-centered care, including revised visiting policies and modalities of information [10].

Since 2001 several French reports are available to examine the evolution of practices. The first one in 2001 described the visiting policies in 92 ICUs [11]. Among them, 97 % had a restrictive visiting policy; only three ICUs reported a 24-h-a-day visiting period. The mean total daily time was 168 min (30–180). The number and the types of visitors were restricted in 95 % and 65 % of ICUs, respectively. Visits of children were permitted in 46 % without any age restriction. In 2009, a second French report launched by the French Society of Intensive Care (SRLF) in 222 ICUs revealed that 58 % of ICUs reported <4 h of visitation time, a 6.7 % of ICUs were open on a 24-h basis, and children visitation without restriction in 3 % of them [12]. The same year, a consensus untitled “For a better life in ICU” was published by the SRLF and French Anesthesiology and Intensive Care Society (SFAR), which provided recommendations about the presence and the role of relatives, staff-family communication, and staff-family information [13]. In 2011, a third report in adult (*n* = 264) and pediatric (*n* = 28) ICUs indicated that 49 % of adult ICUs reported visiting time <4 h a day, 8 % ICUs were open on a 24-h basis, and children visitation without restriction in 12 % of them [14].

In 2014, 5 years later the consensus, our objective was to evaluate how ICUs have included recommendations in their organization of welcoming and of informing families of ICU patients through these six recommendations: (1) free accessibility on a 24-h basis unless patient wishes or special patient care and gowning procedures not systematically used, (2) organization of the presence of children, (3) organization of families’ conferences of information sooner after ICU admission with nurses participation, (4) delivery of an information leaflet, (5) information given and their perception must be written in the chart, and (6) information and organization of families’ participation in care proposal of participation in care.

## Methods

### Participants

#### ICU selection

Among 289 ICU members of the French Society of Intensive Care and French Anesthesiology and Intensive Care Society, an invitation to participate into this survey (2014/02–2014/06) was made to the medical or the nursing head of these units, through a specific phone call. Once their principle agreement was obtained, they received a mail explaining the content of the survey and the possibility to answer either by mail or by a dedicated Web site. These ICUs covered the full French territory (except overseas territories). These ICUs admitted only adult patients. A telephone recall was planned 1 month later. If after 3 months no answer was obtained from the ICU, an e-mail was sent to seek for the causes of no answering among the following proposals: no interest in the survey, not enough time to answer, technical issues, or data insufficiently known.

### Data collection

The questionnaire (Additional file 1) was developed collaboratively between medical researchers (M.G.O., I.V., and A.T.) and some nurses of the French Society of Intensive Care (SRLF). The content was reworded with another group of nurses and physicians (*n* = 12) to ensure its appropriate understanding. The questionnaire included three parts: hospital and ICU characteristics, visiting policies procedures, and management of family information. We did not conduct validity assessments. The study was approved by our institutional board, according to the French law.

#### Hospital and ICU characteristics

The following hospital and ICUs characteristics were collected: regarding the hospital, university, community, or private hospital; and number of beds; regarding the ICU, the structure of the unit (type; number of acute and intermediate beds; number of single beds; number of rooms closed by a door; number of rooms with natural light; presence of a waiting room and family room; number of physicians and fellows; physician-to-patient ratio and nurse-to-patient ratio; shift for nurses and nursing assistants; availability of a psychologist, physiotherapist, social worker, occupational and music therapist; availability of interpret or of religious services.

#### Visiting policies

The following characteristics were reported: time intervals allowed for visit; modification of the time interval in the following cases: end-of-life, request of the patient or the family, clinical worsening, or long stay (>1 month); type and number of persons allowed to visit, gowning procedures, presence of the families or relatives allowed during some specific invasive and noninvasive procedures.

#### Organization of family information

The following data were collected: delivery of an information leaflet, organization of a formal meeting at admission, at discharge, and in end-of-life situations, type of healthcare workers present during these meetings, type of information delivered by nurses throughout the stay, and location of the traceability of information given to families, and implementation of an ICU diary.

Data were described using n (%) and median (interquartile range [IQR]).

## Results

### Characteristics of the ICUs

**Fig. 1:**
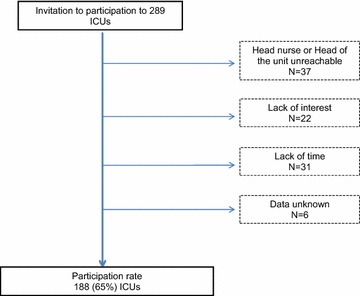
Flowchart of the study

The rate of ICU participation was 188/289 ICUs (65 %). Figure 1 shows the flowchart of the study and the reasons for participation refusal. Characteristics of the 188 participating ICUs are reported in Table 1. Information were reported by medical physicians, especially head of the unit and head nurses in 105/188 (55.9 %) and 83/188 (44.1 %), respectively. Table 2 provides the architectural organization and the available possibilities to welcome families and relatives in the 188 ICUs. Only 118 (62.8 %) of ICUs had a waiting room; 152 (80.9 %) had a dedicated place to inform and communicate with relatives. Families indicated their arrival at ICU door by a combination of possibilities (ring bell, *n* = 103/188 (54.7 %), intercom, *n* = 102 (54.2 %), videophone 36 (19.1 %). Only 2 ICUs had a receptionist to welcome the families. Families were conducted to the patient room by a healthcare worker in 170/188 (90.4 %).

**Table 1:** Characteristics of the participating intensive care units (*n* = 188)

Variables	Data
Hospital	
University hospital	79 (42.0)
Number of beds within the hospital	
<250	29 (15.4)
250–500	62 (32.9)
500–1000	63 (33.5)
>1000	34 (18.0)
ICU	
Medical	35 (18.6)
Surgical	18 (9.6)
Mixed	135 (71.8)
Number of acute beds per unit	12 [10–16]
Number of intermediate beds per unit	6 [4–8]
Number of senior physicians	6 [5–7.5]
Number of junior physicians	4 [2–6]
Day off after duty mandatory for physicians	180 (95.7)
Patient-to-physician ratio during the day	0.31 [0.25–0.41]
Patient-to-nurse ratio during the day	3 [2.5–3]
Patient-to-nurse ratio during the night	3 [2.5–3]
Patient-to-nursing assistant ratio	1.22 [0.88–1.41]
12-h shifts for nurses instead of 8-h shifts	131 (69.6)
Number of psychologists	0 [0–0.2]
Number of physiotherapist	1 [0.5–1.2]
Availability of	
Social worker	82 (43.6)
Occupational therapist	6 (3.2)
Music therapist	1
Access to interpret services	176 (93.6)
Access to religious services	185 (98.4)
Access to ICU follow-up clinic	12 (6.4)

Data are expressed as median [interquartile range] for continuous variables and number (%) for categorical variables

*ICU* intensive care unit

**Table 2:** Architectural characteristics of the 188 intensive care units

Variables	Data
Rate of single-bed room	71.4 [60–100]
Units with rooms closed with a door	175 (93)
Room with natural light	161 (85.6)
Presence of a waiting room	118 (62.7)
Equipped with drinks dispenser	37 (19.6)
Availability of toilets for families	165 (87.7)
Room dedicated to family conferences	152 (80.8)
Family on-site sleep	89 (47.3)
Dedicated room available	10 (5.3)
Possible into the patient room	30 (15.9)
Chair in the waiting room	28 (14.8)
Other possibility	13 (6.9)

Data are expressed as median [interquartile range] for continuous variables and number (%) for categorical variables

#### Visiting policies

**Fig. 2:**
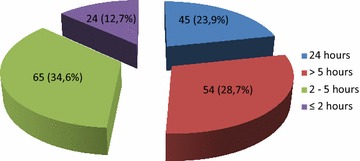
Number of hours offered to presence of relatives in 188 French ICUs

**Fig. 3:**
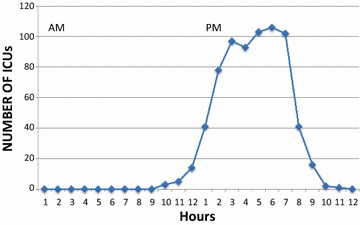
Repartition of presence of relatives in restrictive ICUs with visiting policies

Figures 2 and 3 display visiting policies. Of the 188 ICUs, 45 (23.9 %) were opened on a 24-h-a-day basis. In the remaining ICUs (*n* = 143), the time period allowed for visits was 4.75 ± 1.83 h (median 5 h). The number of slots was 1.5 ± 0.53 (median 1). In 177 (94.1 %), the number of visitors was limited to 2.12 ± 0.37 in the patient room. Visits were strictly limited to families in 15/188 (7.9 %). In all other cases, friends with families could visit the patient. In ICUs where visiting restrictions were reported (*n* = 143), open visiting was allowed for end-of-life situations in 107/143 (74.8 %), when the clinical status worsened (95, 66.4 %), when families requested for increased presence of their relatives (62, 43.3 %), when the patient was conscious (29, 20.3 %), and when the ICU stay lasted more than 1 month (3, 2.1 %). Children were allowed in 164/188 (87.2 %), without restriction on age (from 0 to 18 years old) in 97/164 (59.1 %). Pets were allowed in 4 (2.1 %) ICUs. Personalization of the room with familiar objects of the patient was never allowed in 130/188 (69.1 %) of the ICUs.

#### Gowning procedures

Gowning procedures were imposed to all families in 52/188 (27.6 %) ICUs, whereas 23 (12.3 %) did not impose any to families. In the remaining ICUs (*n* = 113), gowning procedures were imposed in case of multi-resistant bacteria (methicillin-resistant *Staphylococcus aureus* and bacteria producing extended-spectrum beta-lactamase), of *Clostridium difficile*, and of extensively drug-resistant bacteria, in 77/113 (68.1 %), 107/113 (94.6), and 92/113 (81.4 %), respectively. These gowning procedures were overgowns in 162/165 (98.1 %), gloves in 79 (47.8 %), surgical masks in 77 (46.6 %), overshoes in 27 (16.3), and caps in 35 (21.2 %), with various combinations between them displaying all possibilities (data not shown).

#### Information of families

General information of the ICU was given by a nurse or a physician in 162/188 (86.1 %). Special written procedure for informing families existed in 44/188 (23.4 %). Families received an information leaflet in 168/188 (89.3 %). In 11 ICUs (5.8 %) the information leaflet was the solely medium of information without participation of caregivers in the delivery of information. Families’ conferences were scheduled at admission in 149/188 (79.2 %). Discharge conferences were less frequent (60/188, 31.9 %). Nurses and nursing assistants participated in families’ conferences in 133/188 (70.4 %) and 55 (29.2 %), respectively. Residents participated in the delivery of information in 129 (68.6 %), but very few ICUs let these juniors physicians set up the end-of-life conferences (6/188, 3.2 %). A summary of the information delivered to the family was reported in the patient chart by only 111/188 ICUs (59 %). Information over the phone was allowed in 78/188 (41.4 %). Information given by nurses was mainly about comfort symptoms 186/188 (98.9 %), and also disease evolution: 135 (71.8 %), treatments: 66 (35.1 %) or diagnosis: 12 (6.4 %). Of 188, 23 (12.2 %) had an ICU diary: six ICUs for all patients, eight for patients ventilated >48 h, and nine for trauma patients.

#### Presence of families during care

Table 3 displays the presence of families during invasive and noninvasive procedures, which was allowed in very few ICUs. The three procedures where families were most frequently allowed to be present were tracheal aspiration (36/188, 19.1 %), nursing care respecting the need of privacy (51/188, 27.1 %), and rounds with the staff (18/188, 9.6 %). Families participated sometimes in the evaluation of discomfort symptoms and comfort care in 101/188 (53.7 %) and never in 30/188 (16 %).

**Table 3:** Presence of family during invasive and noninvasive procedures in 188 French intensive care units

Variables	Never (and) occasionally *N* = 188	Often (and) always *N* = 188
Introduction of a central venous catheter	186 (98.9)	2 (1.0)
Orotracheal intubation	188 (100)	0
Bronchoscopy	188 (100)	0
Tracheal aspiration	152 (80.5)	36 (19.1)
Echocardiography	175 (93.0)	13 (6.9)
Other types of echography	176 (93.6)	12 (6.3)
Insertion of a gastric tube	181 (96.2)	7 (3.7)
Insertion of a pleural tube	187 (99.4)	1 (0.5)
Cardiac arrest	186 (98.9)	2 (1.0)
Ward rounds	170 (90.4)	18 (9.5)
Nursing care respecting the need of privacy	137 (72.8)	51 (27.1)

Data are expressed as numbers (%)

## Discussion

Thanks to the high participation rate and the diversity of the responding ICUs, our survey on the practices of visiting and information policies in 188 French ICUs provides an accurate overview of families’ management. Recommendations of the consensus “Better life in the ICU” were not implemented in the majority of the ICUs, but progress has been made since the two previous reports in 2001 and 2009. The key findings are that 23.9 % of ICUs applied unrestricted visiting policies; that children were accepted without any barrier of age in 59.1 % of ICUs; that information was delivered to family through structured family meetings on admission in 79.2 % of ICUs and with the participation of nurses in almost 70 % of them. Information delivered to the family was reported in the patient chart by only 111/188 ICUs (59 %). The presence of families during procedures was infrequent.

“Restricting visiting in ICUs is neither caring, compassionate, nor necessary” wrote Berwick and Kotagal [15]. Families of patients are not mere visitors into ICU. Ten years later, and after the publication of international guidelines and position statements made by scientific societies, institutions, and committees, which supported the need for families’ presence based on patient’s preferences and promoted the implementation of an “open ICU model” [16], numerous French ICUs have taken into account the message, and 23.9 % of them open their units on a 24-h-a-day basis. We are facing an evolution of practices in Europe with increased accessibility for families, like in Italy where ICUs have been closed for many years [17, 18] before extending visiting hours [19] and in France [12, 14, 20]. This evolution has probably multiple drivers: expert’s conferences in scientific meetings, positive communication of caregivers in ICUs which have extended these practices [12, 19, 20] and knowledge by the intensivists of the absence of scientific proofs justifying closing ICUs to families. Broad visiting duration was associated with a protective effect on dissatisfaction and on anxiety and depression symptoms [21]. However, there is still a long road to offer to families a true climate of support, as a quarter of the restricted ICUs do not modify visiting policies when death is approaching. This was a surprising and disappointing result compared to other countries like Brazil where only 2.6 % ICUs had a visitation time of 24 h but 99 % of them permitted flexibility in end-of-life situations [22]. This result reflects the fear of caregivers of offering families the psychological support they need. On the opposite, the presence of children without limitation of age was largely favored, even the youngest. Staff fears about visits by children included unproven reasons like worries about infection in both patients and children, disruption into the unit, and deleterious psychological impact [23]. However, tools have been set up for facilitating the children visits. A book containing information on what children would see, hear, and feel can be used to reassure staff and families and to address coping mechanisms [24]. However, few data are available on the impact of children presence in the ICUs [25, 26] and most of the literature is about siblings visiting pediatrics patients. Further studies are required to develop the understanding of children visiting a loved adult. Using systematic gowning procedures for all families in 27 % of ICUs is contrary of French guidelines published in 2009 from the French Society of hygiene [27].

Our results showed that only a few ICUs permitted to families to be present during procedures. This is in opposition with studies reporting family satisfaction, with an improvement of post-traumatic stress disorders, when they participated in comfort care [28], when they were present during brain death evaluation [29] or even during cardiopulmonary resuscitation [30]. Satisfaction and well-being of families in participation of care could be induced by giving a bedside role, by favoring the link with the loved one, and thus having the family feeling useful for the ICU staff. It is time to change, to open our minds and leave our rituals to revamp a different way of providing care, infused with humanity at a high priority level.

Modalities of information are crucial to enhance family satisfaction. Guidelines were recently published to guide staff for providing information to family [31–33]. These guidelines focused on the need of moving from information to communication, of taking time, talking less, avoiding playing the “number games,” and organizing formal meetings (at the third ICU day, on ICU discharge, and for end-of-life situations). We reported that 79 % of the ICUs applied the recommendation of holding a structured meeting at admission. Although we recognize it might be sometimes difficult to set up a structured meeting in the emergency setting when we are caring of the patient, it is necessary to actively approach the family at ICU admission to explain the ICU environment and what is available for diagnosis and treatment. This served to build up a relationship based on trust between healthcare workers and families. Family meetings demonstrated their usefulness not only in end-of-life situations [34–36], but also throughout the ICU stay [37]. Importantly, our report showed that very few ICUs organized a meeting at ICU discharge. However, the transfer from the ICU to a ward unit might be a significantly negative event for some patients and families, generating fear and feelings of abandonment [38]. This meeting is a valuable opportunity for reassuring and informing both patients and families of what will happen in the recovery period. In addition, this meeting could enable a first evaluation of the post-intensive care syndrome [8] before implementing strategies prevention [39]. Very few ICUs applied the recommendation of writing information they gave to families in the chart. This can be the source of difficulties due to contradictory information receiving by the families [32].

Our survey has several limitations. First, 37 % of ICUs denied participating, despite several recalls. We can speculate that these ICUs likely have restricted family’s visiting policies. Second, data were collected from the head nurse or head physician only and on a declarative basis. The ground reality could be slightly different. Third, we did not measure teamwork, safety culture, or healthcare well-being of healthcare workers, although it might influence ICU practices.

In conclusion, this report provides a new insight of the procedures to welcome, manage, and inform families in French ICUs. Although many ICUs do not follow entirely the consensus recommendations, progress has been made since 2001, year of the first report in France. The majority of ICUs has improved their views of welcoming and informing families. We hope that these changes will further progress and that we will keep on opening our minds.

## Additional file

10.1186/s13613-016-0185-x Study questionnaire.
