# Supplementary material for: Reappraisal of visiting policies and procedures of patient’s family information in 188 French ICUs: a report of the Outcomerea Research Group
Source: Ann Intensive Care. 2016 Aug 26;6(1):82. doi: 10.1186/s13613-016-0185-x (PMC4999564; doi:10.1186/s13613-016-0185-x)
Supplement: Supplementary file 1 — 10.1186/s13613-016-0185-x Study questionnaire. [file 13613_2016_185_MOESM1_ESM.docx]

**
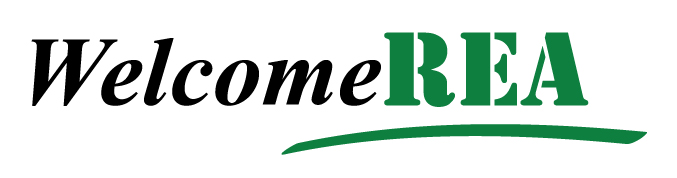
**

**Welcoming and involvement of patient’s family in intensive care units in France**

**Welcome-Rea study**

**Outcomerea research group**

**Mandatory field***

**Identifier (your center number for the study)* :**

**Your position into the ICU***

**Center characteristics :**

Type of hospital * :

University teaching hospital 🗌 General non-teaching hospital 🗌
Private hospital with public hospital missions 🗌 Privately-hold hospital 🗌

Number of acute beds within the hospital *

– <250 🗌

250 à 500 🗌

500 à 1000 🗌

>1000 🗌

Number of beds within the ICU (excluding beds for continuous monitoring)*:

Number of beds for continuous monitoring/intermediate intensive care * :

Type de ICU* : Surgical 🗌 Medical 🗌 Medical-surgical 🗌 Other 🗌

If other, please detail :

**ICU staff characteristics**

Number of equivalent full-time nurses within the ICU*

Number of equivalent full-time physicians within the ICU *

Number of patients per nurse during the day*

Number of patients per nurse during the night *

Number of fellows* Number of medicine trainees*

After-duty day off ? * YES 🗌 NO 🗌

Shift basis for the nurses team *: 12-hour shift 🗌 8-hour shift 🗌 other (please detail) :

Number of equivalent full-time psychologist within the ICU *

Number of equivalent full-time physiotherapist within the ICU *

Number of equivalent full-time nursing assistants within the ICU *

Are there other professionals working within the ICU *

Occupational therapist 🗌 Music therapist 🗌 Social worker 🗌

Are there a translating services available for the patient and patient’s family ?* Yes 🗌 No 🗌

Is it possible to access to different ministers of religion ?* Yes 🗌 No 🗌

**How is the ward structured**

Number of single-bed rooms*

Number of room with 2 beds or more*

à quelle distance du service ?

Does the majority of rooms (>75%) have a door ? * Yes 🗌 No 🗌

Does the majority of rooms (>75%) get natural daylight?* Yes 🗌 No 🗌

How the patients’ family can alert the ward at their arrival ?* (several answers possible):

Ring 🗌 Welcome hostess 🗌 Interphone 🗌 Videophone 🗌 Nothing 🗌

Other (please detail):

Who is in charge of welcoming patient’s family at their arrival* ?

Welcome hostess or equivalent role 🗌

Healthcare workers 🗌

Nobody 🗌

Other 🗌 (please detail) :

Is there a waiting room* ? Yes 🗌 No 🗌

If yes, outside of the ward 🗌 inside the ward 🗌

Is there a beverage dispenser inside the waiting room* ? Yes 🗌 No 🗌

Are there toilets and access to water close to the waiting room* ? Yes 🗌 No 🗌

Can patient’s family sleep on site when requested* ? Yes 🗌 No 🗌

If yes : dedicated room 🗌 a bed set up in patient’s room 🗌

No specific installation 🗌 Other 🗌 (please detail) :

Is there a dedicated room for physicians to meet the patient’s family * ? Yes 🗌 No 🗌

**How patent’s family are welcomed :**

Ward open 24h a day, 7 days a week* Yes 🗌 No 🗌

**If no :**

What are the hours open for visits according to the various periods of time (e.g., 2.00pm – 6.00 pm)

Period 1 Period 2 Period 3 Period 4

Are there any circumstances leading to broader open hours ?* : (several answers possible)

End of life 🗌 Worsening 🗌 Conscious patient 🗌 prolonged stay (> 1 month) 🗌

Upon family request 🗌 Never 🗌 Other 🗌(please detail) : (please detail) :

**Characteristics of people allowed into the ward**

Are there a maximal number of people simultaneously allowed into the patient’s room * Yes 🗌 No 🗌

If yes, how many :

Who can visit the patient *? Family only 🗌 Friends 🗌 both 🗌

Are visits by children allowed *? * Yes 🗌 No 🗌

If yes, if > 15 years 🗌 If > 12 years 🗌 If > 7 years 🗌 Regardless of their age 🗌

Are there gowning procedures set up ?  * (several answers possible)

For visitors of all patients 🗌

Only for visitors of patients carriers of/infected by multi-resistant bacteria (MRSA or BLSE-producing bacteria) 🗌

Only for visitors of patients carriers of/infected by multi-resistant bacteria (MRSA or BLSE-producing bacteria) 🗌

Only for visitors of patients carriers of *Clostridium difficile* 🗌

Not for patient's visitors 🗌

Other 🗌 (please detail) :

If visitors have a gowning procedure to apply, please describe what they have to wear *? (several answers possible)

Gown 🗌 Gloves 🗌 Hairnet 🗌 Mask 🗌 Over-shoes 🗌

At their first visit, are patient's visitors escorted by a member of the staff up to the patient's room* ?

Never 🗌 Sometimes 🗌 Often 🗌 Always 🗌

Can patient's family meet a psychologist into the ward *?

Never 🗌 Sometimes 🗌 Often 🗌 Always 🗌

Do you check family' satisfaction about their welcoming on a regular basis ? * ?

Never 🗌 Sometimes 🗌 Often 🗌 Always 🗌

Are patient's pets allowed into the ward* : Yes 🗌 No 🗌

If yes, do you require the pet's vaccination certificate* ? Yes 🗌 No 🗌

**Family attendance during care procedures**

Into your ward, do you allow family to attend the following care procedures* ? (one answer per procedure)

Catheter insertion Never 🗌 Sometimes 🗌 Often 🗌 Always 🗌

Tracheal intubation Never 🗌 Sometimes 🗌 Often 🗌 Always 🗌

Bronchial fibroscopy Never 🗌 Sometimes 🗌 Often 🗌 Always 🗌

Tracheal aspiration Never 🗌 Sometimes 🗌 Often 🗌 Always 🗌

Cardiac echography Never 🗌 Sometimes 🗌 Often 🗌 Always 🗌

Other echography at bedside Never 🗌 Sometimes 🗌 Often 🗌 Always 🗌

Gastric tube Never 🗌 Sometimes 🗌 Often 🗌 Always 🗌

Pleural drainage Never 🗌 Sometimes 🗌 Often 🗌 Always 🗌

Resuscitation after cardiac arrest Never 🗌 Sometimes 🗌 Often 🗌 Always 🗌

During medical staff rounds Never 🗌 Sometimes 🗌 Often 🗌 Always 🗌

During nursing cares that respect patient's privacy

Never 🗌 Sometimes 🗌 Often 🗌 Always 🗌

Does family participate to patient's discomfort symptoms evaluation *(pain, anxiety, thirst, etc.)

Never 🗌 Sometimes 🗌 Often 🗌 Always 🗌

May the patient's family personalize patients' room with pictures or other personal items* ?

Not allowed 🗌 Authorized 🗌 Supported by staff members 🗌

**How do you inform patient's family ?**

Is there a written protocol for providing information to patient's family* ? Yes 🗌 No 🗌

Does patient's family receive an information leaflet at first visit* ?

Never 🗌 Sometimes 🗌 Often 🗌 Always 🗌

Does patient's family receive general information on intensive care ? (several answers possible)

Yes, by a physician 🗌 Yes, by a nurse 🗌 Yes, by the head of nurses 🗌

Yes, through the information leaflet 🗌 Yes, by another person 🗌 No 🗌

Is a formal meeting held at patient's admission (Day1)* ?

Never 🗌 Sometimes 🗌 Often 🗌 Always 🗌

Is a formal meeting held at patient's discharge from the ICU* ?

Never 🗌 Sometimes 🗌 Often 🗌 Always 🗌

Do nurses take part to meetings with patient's family* ?

Never 🗌 Sometimes 🗌 Often 🗌 Always 🗌

Do nurse assistants take part to meetings with patient's family* ?

Never 🗌 Sometimes 🗌 Often 🗌 Always 🗌

If relatives cannot come to the ICU, do you provide medical information on the phone * ?

Never 🗌 Sometimes 🗌 Often 🗌 Always 🗌

What kind of information nurses provide in your ICU * ? : (several answers possible)

Discomfort symptoms (sleep, pain, mood, etc.) 🗌 Disease evolution 🗌 Diagnosis 🗌

Prognosis 🗌 Treatments 🗌 Treatment limitation 🗌 Death 🗌

**What role has the fellow in information provided to family ?**

Do fellows take part to meetings with patient's family* ?

Never 🗌 Sometimes 🗌 Often 🗌 Always 🗌

Regarding end-of-life meetings, may the fellows conduct them alone *?

Never 🗌 Sometimes 🗌 Often 🗌 Always 🗌

**Traceability of family visits and of information provided to family**

Are family visits reported in patient's chart* ?

Never 🗌 Sometimes 🗌 Often 🗌 Always 🗌

Are information provided to family reported in patient's chart * ?

Never 🗌 Sometimes 🗌 Often 🗌 Always 🗌

Do you offer to patient's family the opportunity to fill in an ICU diary that provides a daily description of patient's ICU stay (and that can also be filled in by staff members) * ?

Yes 🗌 For all patients 🗌 For patients with mechanical ventilation > 48h 🗌

For trauma patients 🗌

No 🗌

**Thank you for your participation to the survey.**

**Dr Maité Garrouste-Orgeas, Dr Alexis Tabah, Dr Isabelle Vinatier, Pr B Misset, Pr JF Timsit**
